# Attitudes, Perceptions and Potential Uptake of Male Circumcision among Older Men in Turkana County, Kenya Using Qualitative Methods

**DOI:** 10.1371/journal.pone.0083998

**Published:** 2014-05-06

**Authors:** Kate Macintyre, Katherine Andrinopoulos, Natome Moses, Marta Bornstein, Athanasius Ochieng, Erin Peacock, Jane Bertrand

**Affiliations:** 1 Aidspan, Nairobi, Kenya; 2 Department of Global Health Systems and Development, Tulane School of Public Health and Tropical Medicine, New Orleans, Louisiana, United States of America; 3 Oxfam-GB, Lodwar, Kenya; 4 Learning for Action, San Francisco, California, United States of America; 5 National AIDS and STD Control Programme, Ministry of Public Health and Sanitation, Nairobi, Kenya; World Health Organization, Switzerland

## Abstract

**Background:**

In many communities, older men (i.e., over 25 years of age) have not come forward for Voluntary Medical Male Circumcision (VMMC) services. Reasons for low demand among this group of men are not well understood, and may vary across geographic and cultural contexts. This paper examines the facilitators and barriers to VMMC demand in Turkana County, Kenya, with a focus on older men. This is one of the regions targeted by the VMMC program in Kenya because the Turkana ethnic group does not traditionally circumcise, and the rates of HIV and STD transmission are high.

**Methods and Findings:**

Twenty focus group discussions and 69 in-depth interviews were conducted with circumcised and uncircumcised men and their partners to elicit their attitudes and perceptions toward male circumcision. The interviews were conducted in urban, peri-urban, and rural communities across Turkana. Our results show that barriers to circumcision include stigma associated with VMMC, the perception of low risk for HIV for older men and their “protection by marriage,” cultural norms, and a lack of health infrastructure. Facilitators include stigma against not being circumcised (since circumcision is associated with modernity), protection against disease including HIV, and cleanliness. It was also noted that older men should adopt the practice to serve as role models to younger men.

**Conclusions:**

Both men and women were generally supportive of VMMC, but overcoming barriers with appropriate communication messages and high quality services will be challenging. The justification of circumcision being a biomedical procedure for protection against HIV will be the most important message for any communication strategy.

## Introduction

Voluntary medical male circumcision (VMMC) is a biomedical intervention with proven efficacy in reducing HIV transmission [1], [2], [3], [4]. The potential impact of VMMC is immense: if 80% coverage is achieved among males 15–49 years in 13 countries of Eastern and Southern Africa by 2025, nearly 4 million infections could be averted [5], [6]. Ability to meet this goal is limited by two challenges: scalability of services and client demand. While headway has been made in some strategies to achieve scale [7], [8], [9], [10], [11], [12], the problem of low demand among older men is persistent across programs. Initial VMMC roll-out attracted mostly boys and men under 25, yet men between 25 and 50 have higher rates of HIV infection and thus need the protection of circumcision as much as the younger generation. Increasing demand among these older clients is a priority in many settings [13].

To date, of the 13 priority countries for VMMC, Kenya has performed the most circumcisions. Forty-nine percent of the 860,000 targeted men (aged 15 to 49) had been circumcised by end of 2012 [14], most of these are amongst the Luo ethnic group in Nyanza and Nairobi. Few circumcisions have been performed among the other non-circumcising groups in Kenya, the Turkana and Teso. Amongst the newly circumcised men and boys (i.e., 430,000), only about 15% were over 25 years [15].

Expansion of VMMC is underway in the Rift Valley Province (RVP), Kenya, where the HIV prevalence is about 7% [16]. The Turkana are the main ethnic group in Turkana County in northern RVP. Estimated circumcision rates among Turkana range from 5 to10% based on hospital data [15], [17]. As in other contexts, early program activities in Turkana suggest cost, pain and fear of the procedure are potential barriers [17], [18], [19]. Additional barriers to VMMC in recent research include tradition and tribal identity, as well as, the idea that circumcision is for children or youth [17]. Perceptions of increased hygiene and sexual satisfaction through circumcision are facilitators of demand [17], [18], [20].

Turkana County is politically, socially, and geographically isolated from other parts of Kenya. The Turkana are mainly pastoralists that have only recently begun to settle in towns. Out of 800,000 residents, 45,368 live in the capital city of Lodwar. Most of the remaining live in scattered communities [21]. Rough, arid terrain and few roads make travel challenging. About 95% of residents live below the poverty line [22], enrollment in primary school is low, and dropout rates among girls are the highest in Kenya with only 7% literacy estimated among women in the County [23]. The health infrastructure is weak: with one district hospital, three sub-district hospitals, eight health centers, and 70 dispensaries, the average distance to a facility is 50 km.

To meet the study’s aim of identifying facilitators and barriers to VMMC among men over 25, we used qualitative research methods including focus group discussions (FGD) and in-depth interviews (IDI). The results are intended to guide new demand creation activities and strategies in Turkana, and in other contexts where VMMC programs are trying to reach geographically dispersed and older clients. The results are also, due to their methodological origins, designed to be hypothesis generating.

## Methods

A cross-sectional study design using qualitative methods was implemented in 2012. We used qualitative methods to elicit attitudes, influences and perceptions which would not have emerged using more quantitative survey methods. As shown in Table 1, 20 FGDs and 64 IDIs were conducted with participants recruited across urban, peri-urban, and rural sites in Turkana.

**Table 1:** Sample of focus group discussions and in-depth interviews by type of participant and research site.

Method	Type of research site			Total
	Urban	Peri-urban	Rural	
**Focus Group Discussions**				
**Circumcised men (25–49)**				
North Turkana	1	1	None*	2
Central Turkana	1	1	None*	2
South Turkana	1	1	None*	2
Lodwar	1	1	None*	2
Subtotal	4	4	0	8
**Uncircumcised men (25–49)**				
North Turkana	0	1	1	2
Central Turkana	1	0	1	2
South Turkana	1	1	1	3
Lodwar	1	0	0	1
Subtotal	3	2	3	8
**Female partners (18–49)**				
North Turkana	0	0	1	1
Central Turkana	0	1	0	1
South Turkana	1	0	0	1
Lodwar	1**	0	0	1
Subtotal	2	1	1	4
**Total FGDs**	**9**	**7**	**4**	**20**
**In-depth interviews**				
**Circumcised men (25–49)**				
North Turkana	3	1	1	5
Central Turkana	4	2	0	6
South Turkana	2	2	0	4
Lodwar	1	1	2	4
Subtotal	10	6	3	19
**Uncircumcised men (25–49)**				
North Turkana	3	1	2	6
Central Turkana	5	2	1	8
South Turkana	1	2	1	4
Lodwar	2	1	0	3
Subtotal	11	6	4	21

Separate FGDs were conducted with older (25–49 years old) uncircumcised and circumcised men, and female partners of either group; and one FGD was held with elder men over 50. IDIs were conducted with participants from the same profile, as well as a few older women and men (over 50). Uncircumcised men were an important group to include to understand perceptions and attitudes to the procedure in this largely uncircumcising traditional ethnic group. Their partners, as well as the sexual partners of the circumcised group are also thought to be an important key group for understanding the current and future demand for circumcision. The main exclusion criterion was age, as no one under 25 years was part of the target group.

The data collection team comprised five Turkana men and one woman, and were led by a researcher from the area. Local expertise, from community-based organization leaders, teachers, and chiefs were asked to refer participants to the study. This was done to ensure a range of outspoken or opinionated individuals took part in the study. Once individuals approached the team, and they had been screened for eligibility (ie age), they were all informed about the purpose and content of the interviews, and were given opportunities to ask questions and to refuse (see the ethics statement below). The refusal rate was 2.7%. These were individuals who decided not to consent to participate after hearing details of the goals and procedures.

### Ethics Statement

The protocol, guides, and consent forms were translated, back-translated, and approved by the Kenya’s national Ethics and Research Committee (ERC) at the Kenyatta National Hospital and University of Nairobi; and by Tulane University’s Institutional Review Board for the Protection of Human Subjects. Most participants gave verbal informed consent, witnessed by two interviewers, or one interviewer and one facilitator. The reason we did this was due to high rates of illiteracy in this region. If a participant could read and write then they gave written consent through signing of the same forms that were read to everyone. Both ethics committees approved this procedure.

All interviews and FGDs were governed by guides, which had been developed by a group of researchers, commented on by a range of experts in the field of circumcision research, and then approved by two ethics committees. The guides were pretested amongst four FGDs and 4 IDIs in Lodwar following a week of training. All the interviews were done in Turkana language, and they were done by members of the same gender, so men interviewed men and the woman interviewed the female partners and led the FGDs with women. The IDIs were held in private spaces and the FGDs (of between 8 and 12 participants) were held in school rooms, clinics or in the open, but without onlookers. On average, the IDIs took about 60 minutes, and the FGDs between 60 and 90 minutes.

All interviews were digitally and manually recorded, and were then transcribed and translated into English by the team, and checked by the supervisor. During field work each team member kept field notes. These were used during the analysis to check details of any special findings that occurred or anecdotal or contextual information that arose during or around the time of the interviews.

Transcripts were reviewed by several of the researchers to develop a coding scheme based on the original aims. This enabled us to analyze the themes arising from those codes using ATLAS.ti 4.2 (Scientific Software Development, Berlin). The team then synthesized key themes to identify barriers and facilitators of demand for VMMC. While some bias (perhaps particularly social acceptability bias) is inevitable, the team reported that the data reflected what they heard and saw as the major reactions of those interviewed. The team were from the ethnic and age group they were interviewing, and the PI did not appear in the field at all. This was to reduce the risk of social acceptability bias, while it may not have eliminated it.

### Data Availability

These data were collected under the Project SEARCH: Research to Prevention (R2P) task order, funded by USAID. As part of this contract, data from all studies must be made publicly available. This will most likely occur through the R2P website (www.jhsph.edu/r2p) at the conclusion of the contract in March 2014.

## Results

Overall, the findings describe barriers and facilitators of demand for VMMC among older men, supply side challenges for increasing VMMC, and recommendations from respondents for improving VMMC services for older men. The results are grouped as follows: cultural significance of circumcision, modernity and disease prevention, circumcision-related stigma, age and marital status, and social influences. Finally, health service factors that influence demand and some of the respondents’ recommendations are summarized. All these themes had been suggested by our reading of the circumcision acceptability literature and other preliminary research done in the area, including consultations with local experts as the guides were developed.

### Cultural Significance of Circumcision

Respondents consistently spoke of circumcision as a practice of other ethnic groups, and as something not aligned with Turkana culture.

*In our culture, we don’t know about ‘the cut’ that is carried out in other tribes that are not Turkana.* (Circumcised man, age 35)

This act of adopting a traditional practice of another culture can carry negative symbolism, as most of Turkana’s territorial neighbors and enemies are from tribes that circumcise men as an initiation rite of passage (e.g., Pokot or Nandi). For some, circumcision signified cultural infidelity and devalued a long-established physical means of marking tribal membership.

*Circumcision is a tradition that is … practiced by tribes like Pokots. So, by them [Turkana] getting circumcised, it will be like leaving their traditions for a different one.* (Partner of uncircumcised man, age 31)

Older Turkana men were apt to see circumcision as disregarding tradition and assimilating to another culture. This perspective among participants was described by a man from a rural area.

*The Turkana men who circumcise are the assimilated group, who stay with the Somalis as workers or their servants. They are influenced by the Somalis culture and beliefs […] Circumcision is a strong departure from the norm and other practices stipulated by the laws of this community.* (Uncircumcised man, age 52)

While these neighboring cultures circumcise men as a rite of passage, the Turkana practice a different ceremony intended to raise men into senior elder status. The significance of this ceremony, called *Asapan*, is sometimes compared to the significance of circumcision in other cultures. As *Asapan* is one of the most important ceremonies that a Turkana man can go through, it is these older men–candidates for *Asapan*–who were most likely to see circumcision as incongruent with their culture.

*Our culture requires that a man should […] undergo Asapan. Circumcision is a strong departure from the norm and other practices stipulated by the laws of this community.* (Uncircumcised man, age 42)

Some men regarded *Asapan* as a divine gift specific to them and distinctive from circumcising cultures.

*On my side, God gave me ‘Asapan’ and circumcision to the other tribes as their culture. What has meaning to Turkana is ‘Asapan’ but not circumcision.* (Uncircumcised man, age 38)

Another idea that emerged from informal discussions during fieldwork gives insight into why some men fear mixing two or more traditions. According to legend, a man who is circumcised and who then goes for *Asapan* may go mad from being caught “between cultures.” The stress of being on two “sides” could potentially drive him insane. Two men told the researchers of a man to whom this had happened (Field notes, April 2012).

While some saw culture as a barrier to VMMC, others said that their cultural practices did not forbid the practice. Several men said circumcision actually has “no meaning” for Turkana. And one can argue that this lack of meaning makes it possible for a culturally-neutral, medical intervention like circumcision. One man said, “[Circumcision] does not mean anything since it is a new thing in Turkana.” Another uncircumcised male advised that circumcision is foreign, but that it can be accepted by Turkana through education:

*“Our people are still not in agreement with this issue of circumcision because it is something foreign …. That is, we did not find our people practicing it before. Therefore, people are still looking at it before they accept it. However, we will come and accept to be circumcised if and only if we are taught well and we understand well the importance of circumcision.”* (Uncircumcised man, age 35)

### Modernity and Disease Prevention

Many circumcised men cited disease prevention as a leading motivating factor in their decision to circumcise. Most who said that VMMC was a preventive measure for HIV approved of the practice. Some used words like “new” or “modern” to describe circumcision and its emergence as a way to fight “new” diseases. They equated HIV as something new or foreign; therefore, a new, foreign approach like VMMC to prevent HIV would be acceptable, regardless of culture.

*What brought circumcision to my community is because of so many issues that have come to the earth… diseases that have come like ‘elepot’ (Gonorrhea) ‘Lokwakel’ (HIV/AIDS). These have made people to see if they can change and follow the recent way of living rather than staying uncircumcised.* (Uncircumcised man, age 30)

While urban dwellers were aware of the protective effects of circumcision, those in rural areas were less able to identify the protection that circumcision offers. These men spoke of the medical benefits of circumcision as a rumor rather than a fact.

### Different Forms of Circumcision-related Stigma

There were negative and positive connotations of stigma related to circumcision discussed by participants. To some, non-circumcision represents a protective notion of cultural assimilation and belonging. This means there is a lot of mocking of individuals (especially in rural areas) who have been circumcised.

There is a common belief that being *uncircumcised* prevents a man from ever being “naked” because he always has a foreskin. Thus, in rural areas where most men do not wear clothes–and indeed walking without “a blanket” is seen as “manly”–the nakedness of being without a foreskin opens men to ridicule.

*Those men who refuse to circumcise mostly fear “Ng’imeny” (ridicule) by either men at the “Alokitoe a Ng’ikiliok” (social gatherings) or women when bathing naked at the water point. While those who circumcise do it because they understand its importance.* (Circumcised man, age 29).

To others, circumcision is understood as part of the necessity of being like others in Kenya. Among these men there is an element of stigma regarding the tradition of *not* circumcising, especially in urban areas, and among those with some education and contact beyond Turkana’s boundaries.

Another stigma related to not circumcising that may prove positive for VMMC programs is that it is increasingly associated with cleanliness, a notion often stemming from religious messages about “cleanliness being close to godliness”. One man said that being uncircumcised made people dirtier.

*Our people carry a lot of dirt in their foreskin after playing sex. This shows that we are still dirty; we have to practice circumcision so that we can be like other people.* (Uncircumcised man, age 35)

Stigmatization goes both ways. While the fear of stigma of being circumcised (and laughed at as not belonging) may prevail as a barrier in more rural areas of Turkana, the stigma from being uncircumcised may be a major catalyst for men and boys becoming circumcised in more urban areas.

### Age and Marital Status

Many of the older men, while expressing positive beliefs about circumcision, often doubted that they would go for the procedure because they were “old” or because they were married and thus felt that it was no longer necessary. The word for “old” in Turkana was used in the interview guides for those over 25 years old – translated directly from English. Though interviewers directed respondents to refer to men 25–49 years of age, interpretation of results must consider the possibility that several respondents may have understood the word “old” to mean elderly, and over the target age of this study.

The idea that marriage is protective against HIV was prevalent. Those who held this notion believed married men were *meant* to be less promiscuous than young, unmarried men. Older age, therefore, may well be a barrier to VMMC because older men are believed to be much less (or even “not at all”) sexually active, and thus less at risk of HIV. Men and women repeatedly said that older men did not need to undergo circumcision “because they were no longer having sex”.

Old men also said that ‘*Lokwakel’* (HIV/AIDS) was not there before. According to them it is the new generation that has brought *‘Lokwakel’*. Thus, circumcision should be for the young, as they are the ones most at risk.

Some expressed concern that circumcision would pose health risks for older men. One woman interviewed stated:

*There is no sense in circumcising [older men], it would even pose some problems to them like taking long to heal and bleeding. To me it is true that circumcision should be done to young energetic men who are still active in playing sex. (*Partner of circumcised man, age 29)

A FGD of women in a peri-urban community offered a different perspective. While most were quick to say circumcision was necessary for young men, these women thought that older men should set a good example.

*Circumcision should start from old men so that young boys see or learn from them because children learn from their parents. And again, none of these men have stopped playing [having] sex. Even the old men are still active in sex.* (FGD with partners of uncircumcised men, peri-urban, central Turkana)

### Social Influences

According to many, the decision to circumcise was often influenced by family and community relationships. A circumcised man narrated his version of how circumcision was gaining acceptance due to the burden of HIV.

*We used to hear our parents say ‘it’s the Pokot, Samburu, Borana and Somali who circumcised.’ But somewhere between there emerged this “Lokwakel” (HIV/AIDS), which wiped out families leaving destitute children. We cried and we decided to circumcise because it was important for us to take care of ourselves and our families.* (Circumcised man, age 37)

Many circumcised respondents, especially the younger men, relayed stories of peer-influence in their decision to get circumcised:

*There was a friend of mine who had decided to go for circumcision and told me, ‘Let’s go and circumcise. How can we continue like this and let other people ridicule us?’ The fear of ridicule from age-mates [already circumcised] influenced our decision.* (Circumcised man, age 35)

Similarly, some uncircumcised men in the more peri-urban areas, said they would be more likely to “go for the cut” if their peers had been circumcised. Others revealed that the decision to get circumcised was a private one. One man said,

*I did not talk to anybody about my decision. I did it alone by going to hospital and afterwards I returned home.* (Circumcised man, age 30)

### Service Delivery Concerns

The remoteness of Turkana has long been a barrier to consistent, equitable health services and this will likely affect the expansion of VMMC. Many expressed fears related to service delivery that could affect demand, including low quality of care; absent, disrespectful or even unqualified clinicians; lack of drugs or equipment; and excessive distance to service delivery sites.

Though rare, stories of VMMC being done improperly were shared. One man described taking a friend to be circumcised and how he was surprised that the man was operated on by a “novice” because he would not pay a bribe at the hospital:

*When I took my friend to be circumcised, we were later shocked to realize he was circumcised by a trainee. The boy bled a lot and he was in great pain and the only experienced clinician who was training the others was demanding Ksh1000, something we believed wasn’t in the policy of the NGO. When we refused to bow to his demands that’s when he directed us to the trainees.* (FGD, Circumcised Men Lodwar)

Supply of VMMC depends on many aspects of a strengthened health system that have yet to adequately reach Turkana. Consistent supply of clean water, electricity, and sterile and private spaces for performing circumcision are often lacking.

### Recommendations to Increase Demand for VMMC Services

Respondents offered suggestions on how to introduce VMMC. Different forms of incentives ranged from cash and food to transport, animals, and medicines. Many said that the program must give sound information on all aspects of the procedure in Turkana language, and that messages should be delivered by local leaders, doctors, or the Ministry of Health.

It was noted that many men could be reached with information and the service itself on the food distribution days that occur across Turkana. Individuals saw opportunity in using NGOs like Oxfam that already have a local presence. Some said peers, especially those already circumcised, should be used as role models to lessen fears associated with circumcision.

## Discussion

Consistent with the objective this study, the findings describe barriers and facilitators for VMMC among older men in Turkana. It is clear that an understanding of the protective effect of circumcision against HIV/STI is as strong facilitator, as has been demonstrated in other research [19], [24], [25]. However, many rural men do not yet see themselves at risk of HIV. Among this group, protection against HIV is not yet a strong argument, since most of those they know who have died of HIV have been urban dwellers and, in many men’s minds, that is where the Turkana who have been circumcised live.

Circumcision is something new–similar to the relative newness of HIV–meriting a reconsideration of this practice. This newness may also be one of the reasons VMMC was perceived as something appropriate for younger men. Similar findings have been found in other contexts [26]. Hygiene in addition to health protection was noted as a benefit of circumcision, again similar to research elsewhere [19], [27]. Messaging around VMMC should highlight the biomedical benefits. For rural dwellers, this should be emphasized with general HIV knowledge. For older men, messaging should focus on the risk of HIV at different life stages, given the perception of “protection due to marriage” and the decreased likelihood of “playing sex” among older men. It is ambiguous if these perceptions match actual risk behavior among this group. Some older men, especially those in rural areas, object to circumcision as it is associated with initiation (to manhood) ceremonies of their enemies. Again, relating male circumcision to a biomedical intervention against HIV will help distance the concept of circumcision as a rite of passage.

In the rural areas, circumcision inferred stigma because it allows the penis to be naked. At the same time, some urban men and women said that non-circumcision also inferred stigma. Among those with access to education, exposure to messages, and contact with HIV, circumcision is associated with modernization, while opposition to circumcision was stronger in the traditional, more rural communities. Messaging should focus on the normality of the circumcised penis: it is not something to be ridiculed; rather, it is done for the man to protect himself and his family against HIV.

Some women noted that elders should serve as role models for the younger generation. Other research has shown women support VMMC overall, often more than men [25]. It is unclear to how women’s ideas may influence men’s decision to circumcise. This issue warrants further investigation into gender dynamics, women’s empowerment, and circumcision across the priority VMMC countries.

Other social influences, including overall community perceptions that VMMC is something that men should do to protect their family and peer influences, were also noted as important, especially among younger men. Social network influences on VMMC uptake are relatively unexplored [28], although the use of peer educators is widespread in VMMC programming. For older men, considerations around appropriate peer-based models may also include other agents such as healers, administrative leaders, teachers, or clinic staff with special training to serve as role models for VMMC. All messaging must be in Turkana language.

Factors noted as barriers in other research studies, including pain and fear, did not surface as important here [18], [19]. This may be due to the focus on older men. Other acceptability studies of VMMC indicate that fear of pain is an issue among young rather than old men [24].

Access to good quality health services to support a VMMC program was described as a major concern. Access issues related to distance may be overcome through transport and food incentives to bring men for a week to stay in urban areas to attend health services. Provision of high quality clinical services through outreach camps to reach many men at once is also recommended.

Consistent with “acceptability” literature [18], [19], [24], [25], [27], these participants felt that circumcision was more appropriate for young rather than older men. Focusing on younger men (aged under 35) would yield more circumcised men as the program is scaled-up, but would also reinforce the idea that VMMC is primarily for youth. This has happened in other contexts, where campaign seasons follow school vacations. Another approach would be to introduce some of the newer techniques to attract older men from programs further along in scale-up, concurrently with the introduction of VMMC in new areas. For example, these may include outreach sites that have all-male staff, offering VMMC through private rather than public services for older men, or giving older men priority in regular clinical settings.

### Study Limitations

Because this is a cross-sectional study, we cannot observe change over time or the dynamic nature of the issues. The use of qualitative methods limits generalizability beyond Turkana. However, idiosyncrasy introduced through an in-depth exploration within the cultural context of Turkana is transferable to other similar settings that are remote, and therefore socially and politically isolated. These methods also allow information to emerge from participants responses, unlike the majority of acceptability studies that force participants to choose from a pre-determined list of barriers and facilitators.

The Turkana word for “old” was used in the interview guides for those over 25 (i.e., translating directly from English). This caused some confusion even when the interviewers repeated the definition of “older” being 25–49 years of age (i.e., middle aged). Several respondents understood the word “old” to mean elderly, so their responses may have been given with men well over 50 in mind. The team did not think this was a major issue across all those interviewed, but it may mean that the findings on “circumcision being only for the young” – are more emphasized in our study results than would have been the case if the translation for “older” had been interpreted as the middle aged (which is not a common concept in Turkana language).

### Conclusion

In a context where communities are physically and food insecure, circumcision is not an urgent priority. Yet many were supportive of VMMC, often positively associating it with modernization, cleanliness, and health benefits. It was noted that older men should be circumcised to serve as role models. The justification of circumcision being protective against HIV is the most important message for any communication strategy, and, in this context, it must clearly be separated from any notion of cultural or traditional circumcision practice. The common idea that older men are less promiscuous and thus less at risk for HIV than younger men, and thus do not need VMMC, must be addressed.
